# Effects of gender, age, family support, and treatment on perceived stress and coping of patients with type 2 diabetes mellitus

**DOI:** 10.1186/1751-0759-8-16

**Published:** 2014-07-15

**Authors:** Yoriko Hara, Mizuho Hisatomi, Hisao Ito, Motoyuki Nakao, Koji Tsuboi, Yoko Ishihara

**Affiliations:** 1 School of Nursing, Kurume University, Higashikushiharamachi 777-1, Kurume, Fukuoka 830-0003, Japan; 2 Tanushimaru Central-clinic, Fukuoka, Japan; 3 Ito Internal Medicine Clinic, Fukuoka, Japan; 4 Department of Public Health, School of Medicine, Kurume University, Fukuoka, Japan; 5 Department of Psychosomatic Medicine, School of Medicine, Toho University, Tokyo, Japan

**Keywords:** Appraisal of Diabetes Scale, Type 2 diabetes mellitus, Stress Coping Inventory, Gender, Spouse

## Abstract

**Background:**

We previously found that the empowerment of patients with type 2 diabetes mellitus can be strongly affected by gender and age in addition to self-managed diet and exercise behaviors and treatment. This study was to examine the effects of gender, age, family support, and treatment on the perceived stress and coping of patients with type 2 diabetes mellitus living with family.

**Methods:**

A survey was conducted of 140 adults with type 2 diabetes mellitus who were living with family. There was no significant difference in hemoglobin A1c (HbA1c) between male and female. Perceived stress and coping were measured with the Japanese version of the Appraisal of Diabetes Scale and the Lazarus Type Stress Coping Inventory. Stepwise regression analysis and path analysis were performed to identify factors that affect the perceived stress and coping of patients.

**Results:**

(1) Perceived stress and coping were strongly affected by gender. (2) Perceived stress and coping were affected by age for males, but perceived stress was not affected by age for females. However, females showed a greater “psychological impact of diabetes” than did males. Females aged between 50 and 69 years engaged in active problem solving, but awareness of diabetes was low. (3) Treatment regimens had an effect on HbA1c for both sexes, and diet therapy affected the awareness of diabetes of males and coping of females. (4) For females, “sense of self-control” was strongly associated with coping, and those who were living with non-spouse family members had a greater psychological impact of diabetes than those living with only their spouse. (5) For males, coping was strongly affected by living with their spouse.

**Conclusions:**

The results suggest that perceived stress, coping, and diet regimen are deeply associated with gender and age and that a male with type 2 diabetes mellitus living with his spouse is strongly dependent on support from the spouse. It is important to take into account gender, age, and family environment to provide patients with an individualized approach to addressing perceived stress and to provide education program for coping that can maximize treatment and maintain better, continuous glycemic control.

## Background

The rapid rise in the number of people suffering from type 2 diabetes mellitus worldwide, in conjunction with increasing life expectancy and changes in lifestyle and social environment, has become an urgent issue from the perspectives of both medical costs and healthcare policy. In Japan, the number of patients suffering from Type 2 diabetes mellitus topped 9.5 million in 2012. Based on the close linkage between this type of diabetes and lifestyle factors, the Japanese government has implemented a “Healthy Japan 21” program focused on Type 2 diabetes mellitus prevention, early detection, and prevention of complications; in addition, it has set targets for reducing the number of patients with type 2 diabetes mellitus through lifestyle improvements based on avoiding the known risk factors as well as through regular checkups and follow-up guidance [1].

Unlike other chronic ailments, the treatment of type 2 diabetes mellitus depends to a great extent on day-to-day self-management of diet, exercise, and other factors; poor glycemic control caused by inadequate self-management can result in complications such as retinopathy, nephropathy, and neuropathy, which markedly reduce the quality of life (QOL) of the patients [2,3]. It has also been reported that restrictions on everyday life imposed by long-term treatment and anxiety over hypoglycemia symptoms can cause chronic stress, further promoting the secretion of corticosteroids and boosting blood sugar levels [4]. These reports point strongly to the importance of self-management of diet, exercise, and other aspects of everyday life by the patient. However, for patients to improve their self-management and maintain it at a high level, they need to be made aware of the emotional burdens of diabetes mellitus and to change their behavior to adapt to those burdens. Recently, the concept of patient empowerment [5], whereby patients are encouraged to gain control over their condition by setting their own blood sugar level targets and taking responsibility for their own behavior, has found widespread acceptance. The same concept has taken hold in Japan as well, with attempts being made to empower patients to change their behavior and to educate them with the aim of maintaining proper glycemic control and a satisfactory QOL.

In an earlier study, we created a Japanese version of the Appraisal of Diabetes Scale (ADS), a self-assessment questionnaire designed to evaluate the patients’ stress awareness and sense of self-control. When we used the ADS with Japanese patients with type 2 diabetes mellitus, the questionnaire was found to consist of 3 subscales, and results for the subscale “Psychological impact of diabetes” in particular were well-correlated with the effects of diet, exercise, oral hypoglycemic agents (OHA), and insulin therapies [6]. Similarly, in another study of Japanese patients with type 2 diabetes for which we created and used a Japanese version of the Diabetes Family Behavior Checklist (DFBC), we found patient glycemic control to be strongly influenced by family attitudes, with poor family support and criticism of patients by family members adversely impacting glycemic control [7]. In a more recent study in which we investigated empowerment in a group of patients with type 2 diabetes mellitus living with their families, we found that patient empowerment was markedly influenced by gender and age as well as by diet/exercise management and treatment method. These results suggest that functional limitations caused by biological and behavioral factors related to gender and age, together with patient emotional factors and environmental factors such as family relationships, contribute in complex ways to diabetes-related stress awareness and coping. For this study, we consequently used a Japanese version of the Lazarus Type Stress Coping Inventory (SCI) with both male and female patients with type 2 diabetes mellitus living with their families to investigate the influence of gender, age, family, and treatment method on stress awareness and coping [8].

## Methods

### Subjects

The subjects were patients who had been diagnosed with type 2 diabetes mellitus at least six months previously; were undergoing therapies that combined OHA, insulin, exercise, and diet therapies; and were living with spouses, children, or other family members. Patients with cognitive disorders or drug-induced/secondary diabetes were excluded. The study was conducted between 2007 and 2011 at six facilities in Fukuoka and Kumamoto Prefectures, including university hospitals, general hospitals, medical centers, and clinics. In all, 140 patients were included in the analysis. Their profiles are summarized in Table 1. There were 73 male and 67 female subjects, with no significant differences observed between the genders with respect to age or duration of disease. With respect to family composition, 94.5% of the male subjects and 65.7% of the female subjects were living with their spouses. As for treatment methods, approximately 50% of the subjects were using diet therapy, 40% exercise, 20% insulin alone, and 70% OHA alone. The mean and standard deviation for the blood glycated hemoglobin (HbA1c) level was 7.09 ± 1.09% for male subjects and 7.47 ± 1.39% for female subjects. Although the levels were higher for females than for males, this difference was not significant.

**Table 1:** Summary of demographic variables

	**Total (n = 140)**	**Male (n = 73)**	**Female (n = 67)**	**P-value**
Age (years)	62.06 ± 11.17	63.20 ± 9.56	60.87 ± 12.66	n.s.
Disease duration (years)	9.90 ± 8.02	9.37 ± 6.90	10.48 ± 9.10	n.s.
Living together with				
(includes multiple answers)				
Spouse	113 (80.7%)	69 (94.5%)	44 (65.7%)	
Sibling (s)	1 (0.7%)	0 (0%)	1 (1.5%)	
Children	82 (58.6%)	34 (46.6%)	48 (71.6%)	
Father/Mother	20 (14.3%)	9 (1.2%)	11 (1.6%)	
Other	2 (1.4%)	1 (0.1%)	1 (0.1%)	
Treatment method (s)				
(includes multiple answers)				
Diet	77 (55.0%)	42 (57.5%)	35 (52.2%)	
Exercise	55 (39.3%)	35 (47.9%)	20 (29.9%)	
Oral hypoglycemic agents	93 (66.4%)	53 (72.6%)	40 (59.7%)	
Insulin	28 (20.0%)	8 (11.0%)	20 (29.9%)	
Oral + Insulin	7 (0.5%)	2 (0.3%)	4 (0.6%)	
HbA1c (%, NGSP*)	7.27 ± 1.23	7.09 ± 1.06	7.47 ± 1.39	n.s.

Data are expressed as mean ± SD.

Statistical analysis was performed by unpaired t-test or Mann–Whitney U test.

n.s.: not significant.

*: NGSP, National Glycohmoglobin Standardization Program.

### Questionnaire structure

We used the Japanese versions of ADS and SCI for patients with type 2 diabetes mellitus to measure their awareness of stress and how they cope with stress, respectively. In addition to items of basic information (age, gender, height, weight, HbA1c, disease duration, treatment method, family composition), the self-report questionnaire used in this study included the 7-item Japanese version of ADS and the 64-item Japanese SCI. We obtained the permission of Carey et al. the authors of the original ADS [4], to produce a Japanese version, which we then checked for reliability and validity [6]. The results of our factor and correlation analyses showed the 7 items of the Japanese ADS to be composed of the following three subscales: “Sense of self-control” (2 items, with higher score being better), “Efforts for symptom management” (1 item, with higher score being better), and “Psychological impact of diabetes” (4 items, with lower score being better). For the SCI, we used a questionnaire commercially available from the Japanese Association of Health Psychology and measured and scored responses according to the methods of implementation and evaluation specified in a common SCI/Ego Aptitude Scale (EAS) manual. The SCI is composed of 10 indicators (Cognitive (Co), Emotional (Em), Problem solving (Pla), Confrontational (Con), Seeking social support (See), Accepting responsibility (Acc),Self-controlling (Sel), Escape-avoidance (Esc),Distancing (Dis), and Positive reappraisal (Pos)), with higher scores indicating stronger tendencies.

### Analytical methods and ethical considerations

Completed questionnaires were all given ID numbers after they were collected, and the data were stored in magnetic media form. The data were statistically analyzed with JMP ver. 10, IBM SPSS ver. 21.0, and IBM Amos ver. 22.0, using t-tests, One-way ANOVA, Mann–Whitney u-test, stepwise multiple regression, and path analysis, with the significance criterion set at P < 0.05. This study was approved in advance by the Clinical Ethics Committee of Kurume University. The objectives, methods, and other details of the study were explained to the study subjects and their written informed consent was obtained.

## Results

### The relationship between stress awareness/coping and gender, age, family, and treatment method

**Figure 1:**
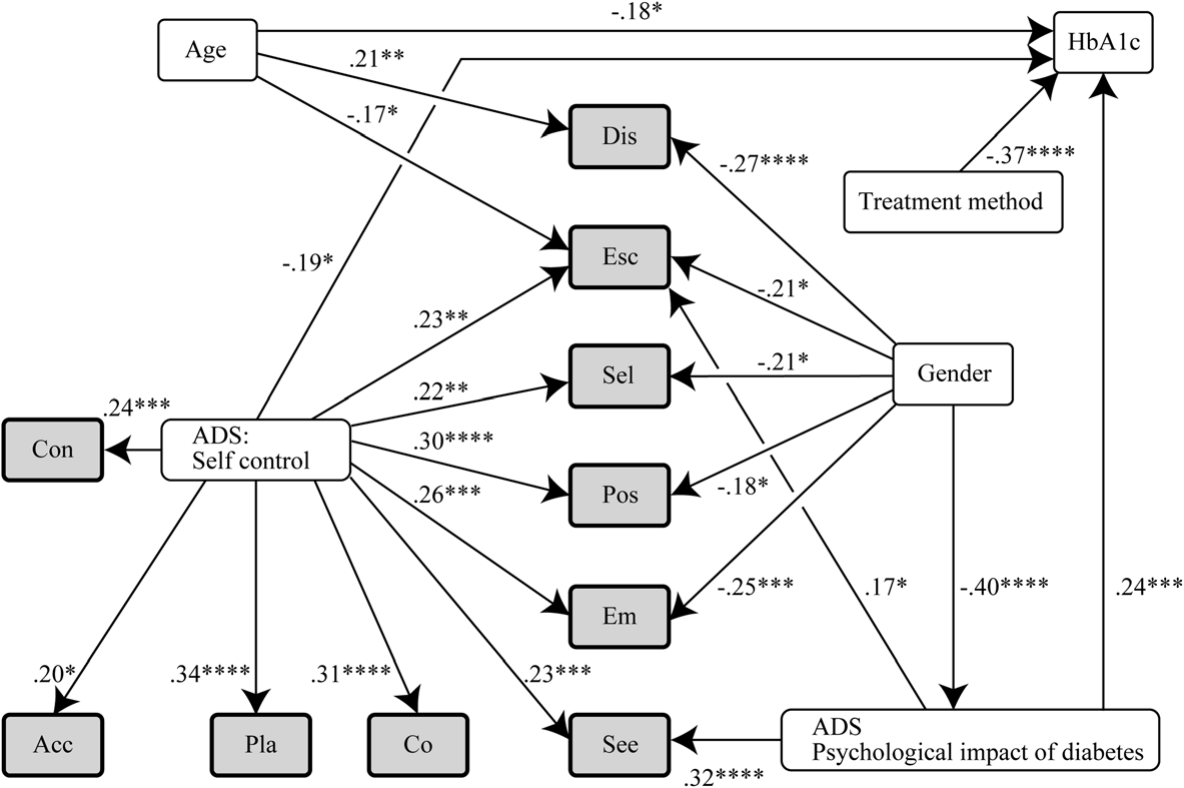
**Analysis of factors that influence coping.** Stepwise multiple regression analysis and path analysis were used to model the factors that influence the 10 SCI indicators. Females were assigned a value of 0 and males a value of 1. For the treatment methods, patients undergoing either diet or exercise were assigned a value of 1, patients undergoing both diet and exercise a value of 2, patients taking OHAs irrespective of diet and exercise therapy a value of 3, patients receiving insulin injections irrespective of diet and exercise therapy a value of 4, and patients receiving both OHAs and insulin injections a value of 5. The figures above the arrowed lines are standardized coefficients. *: P < 0.05, **: P < 0.01, ***: P < 0.005, ****: P < 0.001. Abbreviations for SCI indicators, Pla: Problem solving, Con: Confrontational, See: Seeking social support, Acc: Accepting responsibility, Sel: Self-controlling, Esc: Escape-avoidance, Dis: Distancing, Pos: Positive reappraisal, Co: Cognitive, Em: Emotional.

Figure 1 shows the results of path analysis carried out by stepwise multiple regressions of the data for all study subjects, together with the standardized coefficients. Gender was strongly correlated with the ADS “Psychological impact of diabetes” subscale and with the SCI indicators Dis, Esc, Sel, Pos, and Em; age with HbA1c level and with SCI indicators Dis and Esc; and treatment method with HbA1c. However, family relationships were weakly correlated with the three ADS subscales and SCI indicators. The strong correlations between the ADS “Sense of self-control” subscale and all SCI indicators except Dis suggest that the self-assessments of execution of self-control and its efficacy were clearly measured.

### The influence of gender on stress awareness and coping

Because of the strong linkage between gender and stress awareness/coping, we compared scores for the three ADS subscales and 10 SCI indicators according to gender (Table 2). Scores for the ADS “Psychological impact of diabetes” subscale were significantly higher for females than for males, suggesting that female patients feel greater stress than male patients. Females also showed markedly higher scores than males for the SCI indicators Sel, Esc, Dis, and Em. This suggests that although females feel greater stress than males, they are more reluctant than males to cope with stress.

**Table 2:** Comparison of ADS and SCI scores according to gender

		**Male (n = 73)**	**Female (n = 67)**
ADS			
	Sense of self-control^1)^	6.18 ± 1.02	5.91 ± 1.12
	Psychological impact of diabetes^2)^	9.18 ± 2.80^***^	11.55 ± 2.91
	Efforts for symptom management^3)^	2.32 ± 0.83	2.33 ± 0.75
SCI			
	Pla (Problem solving)	6.92 ± 3.75	6.79 ± 3.99
	Con (Confrontational)	5.16 ± 3.35	4.70 ± 3.15
	See (Seeking social support)	4.15 ± 3.46	4.85 ± 3.27
	Acc (Accepting responsibility)	6.85 ± 4.12	7.09 ± 3.74
	Sel (Self-controlling)	6.07 ± 3.52^*^	7.61 ± 3.73
	Esc (Escape-avoidance)	3.99 ± 2.62^**^	5.58 ± 3.14
	Dis (Distancing)	5.48 ± 3.48^**^	7.48 ± 4.14
	Pos (Positive reappraisal)	6.66 ± 4.33	8.00 ± 4.02
	Co (Cognitive)	24.10 ± 12.56	25.16 ± 12.11
	Em (Emotional)	21.18 ± 11.52^**^	26.97 ± 12.79

^1)^, ^3)^: Higher scores are better; ^2)^: Lower scores are better.

Data are expressed as mean ± SD.

Statistical analysis was performed by unpaired t-test.

Male vs. Female, *: P < 0.05, **: P < 0.01, ***: P < 0.001.

### The influence of age on stress awareness and coping

Table 3 shows that scores for the ADS “Sense of self-control” and “Efforts for symptom management” subscales did not show any influence of age for either males or females, but the scores for the ADS “Psychological impact of diabetes” subscale showed a tendency to decrease with the age of males, suggesting that males tend to be less affected psychologically as they grow older. However, because the standard deviations were large, this change was not significant. For females, the ADS “Psychological impact of diabetes” subscale scores showed no age influence, although the scores for this subscale were higher for females than for males across all age groups. This suggests that female patients of all ages suffer greater psychological stress from type 2 diabetes mellitus than do males. Among the SCI indicators, Sel and Esc scores showed a significant age influence for both genders, whereas Acc, Dis, Pos, and Em showed a significant age influence only for females. Most of the scores for indicators influenced by age showed U-shaped variation, being higher for patients 49 and under and for patients 70 and over, and lower in the 50–69 years age range.

**Table 3:** Comparison of ADS and SCI scores according to age group

	**ADS**								**SCI**					
	**Sense of self-control**^**1)**^				**Psychological impact of diabetes**^**2)**^		**Efforts for symptom management**^**3)**^		**Pla**		**Con**		**See**	
**Years**	**Male (n = 73)**	**n**	**Female (n = 65)**	**n**	**Male**	**Female**	**Male**	**Female**	**Male**	**Female**	**Male**	**Female**	**Male**	**Female**
40-49	6.00 ± 1.15	7	5.57 ± 0.76	13	11.00 ± 1.63	11.72 ± 3.81	2.57 ± 0.79	2.64 ± 0.74	7.57 ± 2.99	7.43 ± 3.06	8.29 ± 3.30	6.00 ± 2.94	6.14 ± 3.76	5.43 ± 3.99
50-59	6.06 ± 0.85	16	5.58 ± 0.90	12	9.88 ± 2.68	11.50 ± 2.78	2.38 ± 1.02	2.17 ± 1.11	6.81 ± 3.69	6.17 ± 2.86	4.69 ± 2.63	4.42 ± 2.68	4.75 ± 3.68	5.08 ± 2.54
60-69	6.26 ± 1.03	31	6.15 ± 1.18	19	8.90 ± 2.95	11.65 ± 3.10	2.29 ± 0.74	2.20 ± 0.52	6.61 ± 3.76	6.20 ± 4.38	4.58 ± 2.94	4.00 ± 3.01	3.87 ± 3.49	4.15 ± 2.68
70-79	6.21 ± 1.13	19	6.10 ± 1.34	21	8.37 ± 2.73	11.38 ± 2.27	2.21 ± 0.85	2.33 ± 0.66	7.26 ± 4.24	7.29 ± 4.77	5.37 ± 4.07	4.67 ± 3.58	3.37 ± 2.99	5.00 ± 3.71
	**SCI**													
	**Acc**		**Sel**		**Esc**		**Dis**		**Pos**		**Co**		**Em**	
**Years**	**Male**	Female*	Male*	Female*	Male**	Female*	**Male**	Female**	**Male**	Female**	**Male**	**Female**	**Male**	Female*
40-49	7.71 ± 4.86	7.86 ± 2.60	9.00 ± 2.31	7.79 ± 2.86	7.00 ± 3.06	7.00 ± 3.37	5.00 ± 2.65	7.29 ± 3.91	6.71 ± 4.54	9.07 ± 3.95	29.43 ± 12.71	27.21 ± 11.00	28.00 ± 9.93	30.71 ± 11.44
50-59	6.25 ± 4.16	5.67 ± 3.17	5.31 ± 3.09	7.58 ± 2.94	4.19 ± 2.48	4.42 ± 2.64	5.25 ± 2.54	5.67 ± 3.23	7.13 ± 3.98	6.83 ± 1.99	23.06 ± 12.18	22.50 ± 7.47	21.31 ± 10.71	23.33 ± 9.08
60-69	6.19 ± 3.75	5.75 ± 4.30	5.29 ± 3.30	5.70 ± 3.56	3.48 ± 2.59	4.45 ± 2.84	4.87 ± 3.34	6.05 ± 4.11	6.26 ± 4.45	6.05 ± 3.62	22.71 ± 12.19	21.30 ± 12.64	18.45 ± 11.44	21.05 ± 12.61
70-79	8.11 ± 4.36	8.67 ± 3.55	6.89 ± 4.01	9.33 ± 4.14	3.53 ± 1.95	6.38 ± 3.09	6.84 ± 4.40	10.00 ± 3.74	6.89 ± 4.63	9.81 ± 4.42	25.26 ± 13.76	29.00 ± 13.61	23.00 ± 12.22	32.19 ± 13.30

^1)^, ^3)^: Higher scores are better; ^2)^: Lower scores are better.

Data are expressed as mean ± SD.

Statistical analysis was performed by one-way ANOVA. *: P <0.05, **: P <0.01 (age groups in each gender).

Pla: Problem solving, Con: Confrontational, See: Seeking social support, Acc: Accepting responsibility, Sel: Self-controllling, Esc: Escape-avoidance,

Dis: Distancing, Pos: Positive reappraisal, Co: Cognitive, Em: Emotional.

### The influence of treatment method on stress awareness and coping

We compared the influence of four treatment methods (diet, exercise, OHA, and insulin) on stress awareness and coping (Table 4). A comparison of therapy and non-therapy groups for each treatment method grouped by gender showed that among males, scores were significantly higher than for non-therapy groups on the ADS “Sense of self-control” subscale for those undergoing diet therapy and on the ADS “Efforts for symptom management” subscale for those undergoing insulin therapy. With respect to the influence of therapies on the 10 SCI indicators, no influence was found in any indicator among males, although among females, Pla, Sel, Pos, and Co scores for those undergoing diet therapy and the Con score for those undergoing insulin therapy were significantly higher than for each of the non-therapy groups. These results suggest that female patients on diet or insulin therapies tend to be more methodical and demonstrate greater self-control and motivation to tackle their problems.

**Table 4:** Comparison of ADS and SCI scores according to treatment method

**Treatment**		**Diet**				**Exercise**				**Oral hypoglycemic agents**				**Insulin**			
		**Male**		**Female**		**Male**		**Female**		**Male**		**Female**		**Male**		**Female**	
		**Yes n = 42**	**No n = 31**	**Yes n = 35**	**No n = 32**	**Yes n = 35**	**No n = 38**	**Yes n = 20**	**No n = 47**	**Yes n = 53**	**No n = 20**	**Yes n = 40**	**No n = 27**	**Yes n = 8**	**No n = 65**	**Yes n = 20**	**No n = 46**
ADS																	
Sense of self-control^1)^		6.50 ± 0.97**	5.74 ± 0.93	5.97 ± 1.04	5.84 ± 1.22	6.37 ± 0.91	6.00 ± 1.09	6.15 ± 1.14	5.81 ± 1.12	6.19 ± 1.11	6.15 ± 0.75	5.90 ± 1.06	5.93 ± 1.24	5.88 ± 0.35	6.22 ± 1.07	6.15 ± 1.35	5.81 ± 1.01
Psychological impact of diabetes^2)^		9.00 ± 3.00	9.42 ± 2.53	11.43 ± 2.88	11.69 ± 2.99	9.34 ± 2.80	9.03 ± 2.83	12.25 ± 2.83	11.26 ± 2.93	8.94 ± 3.03	9.80 ± 1.99	11.58 ± 2.85	11.52 ± 3.06	9.75 ± 1.28	9.11 ± 2.93	12.50 ± 3.46	11.15 ± 2.59
Efforts for symptom management^3)^		2.29 ± 0.83	2.35 ± 0.84	2.34 ± 0.76	2.31 ± 0.74	2.31 ± 0.80	2.32 ± 0.87	2.15 ± 0.49	2.40 ± 0.83	2.21 ± 0.82	2.60 ± 0.82	2.30 ± 0.76	2.37 ± 0.74	2.88 ± 0.83*	2.25 ± 0.81	2.20 ± 1.01	2.38 ± 0.61
SCI	Pla	7.14 ± 3.96	6.61 ± 3.49	8.20 ± 4.00**	5.25 ± 3.43	6.91 ± 3.72	6.92 ± 3.83	8.25 ± 3.63	6.17 ± 4.02	7.09 ± 3.88	6.45 ± 3.44	6.38 ± 4.00	7.41 ± 3.97	6.13 ± 4.36	7.02 ± 3.70	7.35 ± 4.65	6.55 ± 3.71
	Con	4.79 ± 3.34	5.68 ± 3.36	5.37 ± 3.16	3.97 ± 3.01	4.51 ± 2.76	5.76 ± 3.78	5.00 ± 2.77	4.57 ± 3.31	5.43 ± 3.59	4.45 ± 2.58	4.45 ± 3.31	5.07 ± 2.91	4.88 ± 3.60	5.20 ± 3.35	5.90 ± 3.52*	4.19 ± 2.86
	See	4.45 ± 3.85	3.74 ± 2.86	5.09 ± 3.23	4.59 ± 3.35	3.89 ± 3.48	4.39 ± 3.47	5.30 ± 3.44	4.66 ± 3.22	4.26 ± 3.62	3.85 ± 3.05	4.35 ± 3.29	5.59 ± 3.15	4.00 ± 2.33	4.17 ± 3.59	5.10 ± 2.79	4.74 ± 3.48
	Acc	7.05 ± 3.94	6.58 ± 4.39	7.57 ± 3.88	6.56 ± 3.56	7.29 ± 3.77	6.45 ± 4.42	8.05 ± 3.94	6.68 ± 3.62	6.87 ± 4.28	6.80 ± 3.75	6.68 ± 3.93	7.70 ± 3.42	5.25 ± 3.92	7.05 ± 4.12	6.75 ± 4.18	7.23 ± 3.58
	Sel	6.00 ± 3.72	6.16 ± 3.30	8.57 ± 3.70*	6.56 ± 3.53	5.63 ± 3.03	6.47 ± 3.92	8.55 ± 3.09	7.21 ± 3.94	6.30 ± 3.60	5.45 ± 3.32	7.25 ± 3.81	8.15 ± 3.61	5.00 ± 4.31	6.20 ± 3.43	8.10 ± 4.01	7.40 ± 3.63
	Esc	3.57 ± 2.42	4.55 ± 2.81	5.94 ± 3.08	5.19 ± 3.22	3.57 ± 2.38	4.37 ± 2.80	6.45 ± 3.24	5.21 ± 3.06	4.04 ± 2.75	3.85 ± 2.30	5.25 ± 3.04	6.07 ± 3.28	3.50 ± 2.27	4.05 ± 2.67	5.70 ± 3.16	5.53 ± 3.17
	Dis	5.21 ± 3.63	5.84 ± 3.30	7.94 ± 4.15	6.97 ± 4.12	5.20 ± 3.39	5.74 ± 3.59	8.40 ± 3.66	7.09 ± 4.30	5.53 ± 3.59	5.35 ± 3.25	7.13 ± 4.29	8.00 ± 3.92	5.00 ± 3.96	5.54 ± 3.45	8.30 ± 4.07	7.13 ± 4.16
	Pos	6.57 ± 4.66	6.77 ± 3.91	9.03 ± 3.82*	6.88 ± 3.98	6.80 ± 4.41	6.53 ± 4.31	9.65 ± 3.62*	7.30 ± 4.00	6.77 ± 4.49	6.35 ± 3.96	7.58 ± 4.23	8.63 ± 3.66	6.25 ± 4.53	6.71 ± 4.34	8.55 ± 4.19	7.77 ± 3.96
	Con	24.71 ± 12.78	23.26 ± 12.42	28.43 ± 11.60*	21.59 ± 11.81	23.97 ± 11.60	24.21 ± 13.54	29.25 ± 11.00	23.43 ± 12.25	24.60 ± 13.09	22.75 ± 11.25	23.75 ± 12.72	27.26 ± 11.03	21.25 ± 13.79	24.45 ± 12.48	26.15 ± 12.28	24.74 ± 12.14
	Em	20.07 ± 11.86	22.68 ± 11.05	29.34 ± 12.33	24.38 ± 12.97	19.83 ± 10.07	22.42 ± 12.71	30.45 ± 10.89	25.49 ± 13.35	21.70 ± 12.22	19.80 ± 9.54	25.35 ± 12.83	29.37 ± 12.58	18.75 ± 12.60	21.48 ± 11.45	29.60 ± 14.07	25.85 ± 12.19

^1)^, ^3)^: Higher scores are better; ^2)^: Lower scores are better.

Data are expressed as mean ± SD.

Statistical analysis was performed by unpaired t-test. Treatment vs. Non-treatment *: P < 0.05, **: P < 0.01.

Pla: Problem solving, Con: Confrontational, See: Seeking social support, Acc: Accepting responsibility, Sel: Self-controlling, Esc: Escape-avoidance,

Dis: Distancing, Pos: Positive reappraisal, Co: Cognitive, Em: Emotional.

### The influence of cohabiting family on stress and coping

**Figure 2:**
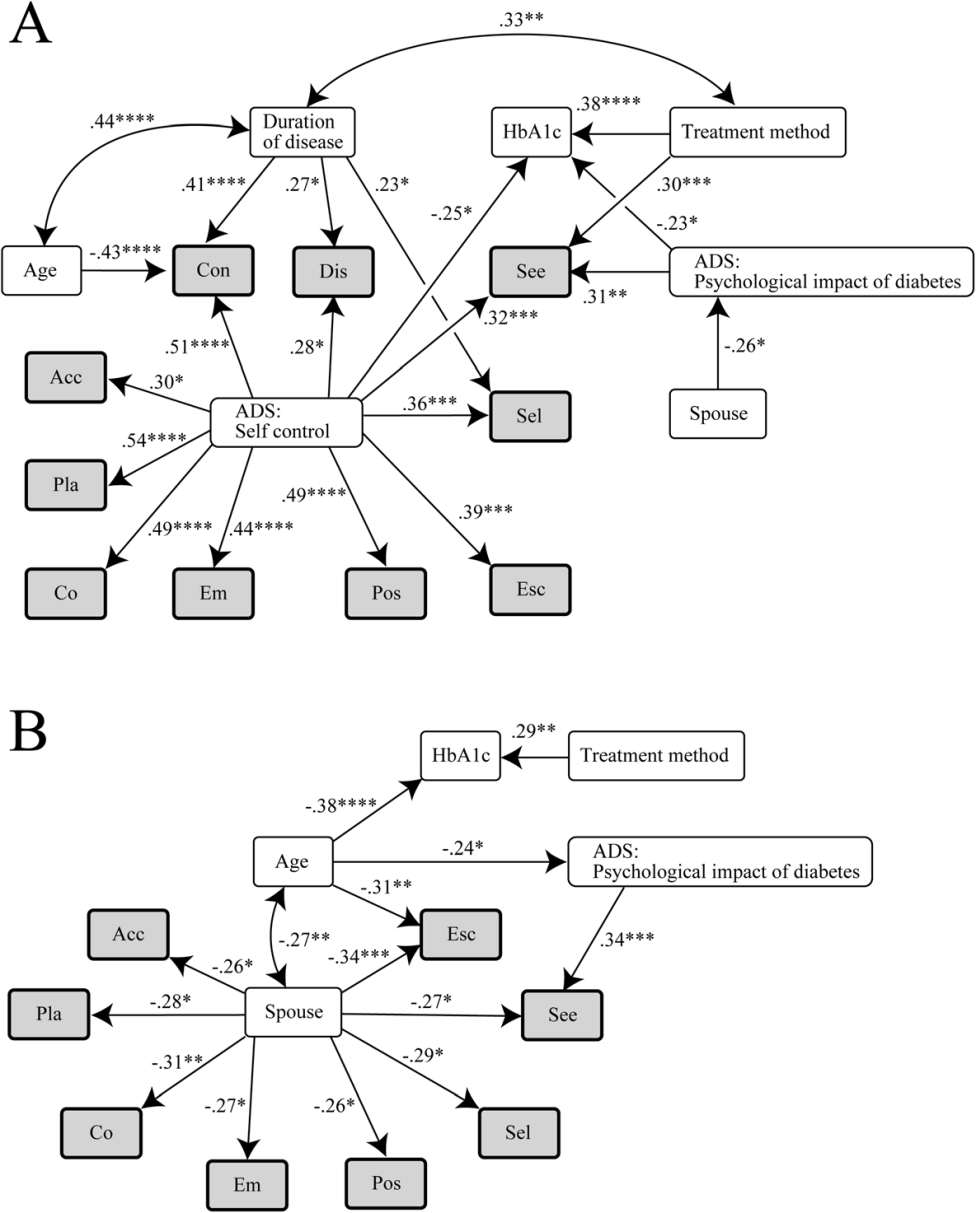
**Analysis of factors that influence coping.** Stepwise multiple regression analysis and path analysis were used to model the factors that influence the 10 SCI indicators separately for females **(A)** and males **(B)**. For the treatment methods, patients undergoing either diet or exercise were assigned a value of 1, patients undergoing both diet and exercise a value of 2, patients taking OHAs irrespective of diet and exercise therapy a value of 3, patients receiving insulin injections irrespective of diet and exercise therapy a value of 4, and patients receiving both OHAs and insulin injections a value of 5. Patients not living with spouses were assigned a value of 0 and those living with spouses a value of 1. The figures above the arrowed lines are standardized coefficients and those above the two-headed arrowed lines are correlation coefficients between the two variables. *: P < 0.05, **: P < 0.01, ***: P < 0.005, ****: P < 0.001. Abbreviations for SCI indicators, Pla: Problem solving, Con: Confrontational, See: Seeking social support, Acc: Accepting responsibility, Sel: Self-controllling, Esc: Escape-avoidance, Dis: Distancing, Pos: Positive reappraisal, Co: Cognitive, Em: Emotional.

Figure 2 shows the results of the path analyses carried out for both genders. The upper panel A shows the results for females and the lower panel B for males. Among the females, the ADS “Sense of self-control” subscale showed strong linkage with all SCI indicators. However, the treatment method and ADS “Psychological impact of diabetes” subscale showed only links with the SCI indicator See and the HbA1c level. Disease duration was also correlated with the SCI indicators Con, Dis, and Sel among females, the first being particularly strong. Among males, the presence of a spouse was strongly correlated with eight SCI indicators, whereas the ADS “Psychological impact of diabetes” subscale was correlated only with the SCI indicator See. Table 5 shows the mean values and standard deviations for each of the ADS subscales and SCI indicators grouped by gender. A comparison of the spouse groups with the no-spouse groups shows that scores for the ADS “Psychological impact of diabetes” subscale tended to be lower for females with spouses, whereas the SCI scores showed no clear influence. In contrast, for males there was no significant difference in the ADS subscale scores between the spouse/no-spouse groups, whereas the scores for all SCI indicators were lower for males with spouses compared with males without spouses, with the differences being significant for all indicators except Con and Dis.

**Table 5:** Comparison of ADS and SCI scores according to cohabiting family

		**Male**		**Female**	
		**With spouse (n = 69)**	**Without spouse (n = 4)**	**With spouse (n = 44)**	**Without spouse (n = 23)**
ADS					
	Sense of self-control^1)^	6.13 ± 0.98	7.00 ± 1.41	5.75 ± 1.01	6.22 ± 1.28
	Psychological impact of diabetes^2)^	9.22 ± 2.85	8.50 ± 1.73	10.89 ± 2.91^**^	12.83 ± 2.52
	Efforts for symptom management^3)^	2.32 ± 0.83	2.25 ± 0.96	2.30 ± 0.67	2.39 ± 0.89
SCI					
	Pla (Problem solving)	6.67 ± 3.62^*^	11.25 ± 3.77	6.64 ± 4.08	7.09 ± 3.90
	Con (Confrontational)	5.03 ± 3.26	7.50 ± 4.65	4.66 ± 3.12	4.78 ± 3.27
	See (Seeking social support)	3.94 ± 3.41^*^	7.75 ± 2.22	4.41 ± 3.26	5.70 ± 3.20
	Acc (Accepting responsibility)	6.59 ± 4.02^*^	11.25 ± 3.59	7.55 ± 3.76	6.22 ± 3.63
	Sel (Self-controlling)	5.83 ± 3.38^*^	10.25 ± 3.77	7.77 ± 3.56	7.30 ± 4.12
	Esc (Escape-avoidance)	3.83 ± 2.51^*^	6.75 ± 3.30	5.66 ± 3.08	5.44 ± 3.33
	Dis (Distancing)	5.32 ± 3.38	8.25 ± 4.50	7.64 ± 4.24	7.17 ± 4.00
	Pos (Positive reappraisal)	6.39 ± 4.24^*^	11.25 ± 3.59	8.00 ± 3.84	8.00 ± 4.42
	Co (Cognitive)	23.17 ± 12.09^**^	40.00 ± 10.95	24.82 ± 12.11	25.83 ± 12.36
	Em (Emotional)	20.42 ± 10.90^*^	34.25 ± 15.82	27.55 ± 12.50	25.87 ± 13.54

^1)^, ^3)^: Higher scores are better; ^2)^: Lower scores are better.

Data are expressed as mean ± SD.

Statistical analysis was performed by unpaired t-test.

With spouse vs. Without spouse *: P < 0.05, **: P < 0.01.

## Discussion

We used the ADS questionnaire developed by Carey et al. and the Lazarus Type SCI questionnaire to examine how patients with type 2 diabetes mellitus appraise their own awareness of stress stemming from diabetes mellitus and how they cope with that stress, respectively. The results of this study enabled us to draw the following conclusions: (1) stress awareness and coping are greatly influenced by gender; (2) aging influences the stress awareness and coping of males and has no influence on stress awareness of females, although females of all ages suffer greater “psychological impact from diabetes” than do males, with those in the 50 to 69 years age group being motivated to actively address their problems but possessing low self-awareness; (3) The treatment regimen has an effect on the HbA1c of both genders, and diet therapy affects the awareness of diabetes of males and the coping of female; (4) females show a strong correlation between “Sense of self-control” and coping, and “Psychological impact of diabetes” is higher for females living with non-spouses than for those living with spouses; (5) living with a spouse has a strong positive influence on the ability of males to cope with stress.

Gender differences in stress awareness and coping have been reported to involve social, innate, and circumstantial factors [9,10]. In this study as well, we found gender differences in the ADS “Psychological impact of diabetes” subscale that reflect the degree of stress awareness and in SCI indicators that reflect the ability to cope emotionally with diabetes mellitus and respond flexibly to circumstances. The significantly higher scores of females compared with males in these self-appraisals suggest that female patients feel greater stress from diabetes mellitus than do males and that their cautious approach to dealing with the disease tends to make them more reluctant to take action and less flexible in their responses. It seems likely that this behavior is strongly influenced by innate factors. A number of studies have been conducted on gender differences in patients with type 2 diabetes mellitus [11-13]. Chiu et al. also used biological, behavioral, and psychosocial indicators to study these gender differences and found that whereas females excelled in self-management of diet and blood sugar determination, they did not do as well as males in exercise activities, appreciation of the importance of glycemic control, self-efficacy, coping, depressive symptoms, and family support, suggesting that female-specific biological and behavioral factors contribute directly to functional limitations [14]. Accordingly, it seems likely that any initiatives to improve the psychological satisfaction levels of female patients and their self-management through glycemic control and stress coping will require either external support or careful attention to these female-specific biological and behavioral functional limitations.

In the present study, a gender difference in the relationship between age and stress awareness/coping was observed, with “Psychological impact of diabetes” declining with the age of males. Thus, males tended to suffer less stress from diabetes mellitus as they grew older (Table 3 and Figure 2B). Males were also suggested to be more eager to tackle their ailment as they aged, despite the fact that their self-control of coping showed a tendency to decline with age. This may be due to the fact that most of the male subjects of this study were living with their spouses. In Japan, males tend to retire from work between the ages of 60 and 65. They then have more time and closer engagement with family members. Diet and exercise self-management may have become easier; consequently, it was assumed that males became less prone to suffer psychologically and more motivated to tackle the disease as they grew older. Females, on the other hand, showed no age influence where “Psychological impact of diabetes” was concerned, but proved to suffer greater psychological impacts than males across all age groups. Additionally, where coping is concerned, female aged 50 to 69 were more motivated than those of other ages to tackle their disease, but tended to show less self-control, self-awareness, and understanding of their own role in coping with diabetes mellitus. As mentioned above, this was presumably a result of the influence of female-specific biological and behavioral functional limitations. No detailed studies on this aspect have as yet been carried out in Japan: further research needs to be conducted on the combined impacts of gender and aging.

The path analysis conducted to examine the relationship between the treatment method and stress awareness/coping of patients with type 2 diabetes mellitus revealed correlations between the treatment method and HbA1c level as well as the desire for social support in coping with stress (SCI See) only for females. A comparison of the ADS subscale and SCI indicator scores of the therapy and non-therapy groups for each treatment method grouped by gender showed that among males the scores on the ADS “Sense of self-control” subscale were significantly higher for the therapy than the non-therapy group only for those undergoing diet therapy, whereas females receiving diet therapy earned significantly higher coping scores than did non-therapy groups for self-control, positive reappraisal, and being methodical and proactive. These results show that most of the males undergoing diet therapy take the attitude that they are realistically controlling their diabetes mellitus through diet, whereas most females receiving diet therapy tend to view such therapy as being essential to coping with type 2 diabetes mellitus. This strongly suggests a gender difference in stress awareness/coping with respect to diet self-management. Other treatment methods, however, showed few such trends or clear impacts on stress awareness/coping. There appear to be no reports to date that have used ADS and Lazarus Type SCI questionnaires with patients with type 2 diabetes mellitus that compare treatment methods and stress awareness/coping. Welch et al. have used the Problem Areas in Diabetes scale and other tools to compare the effect of insulin therapy, OHA therapy, and diet therapy on coping and emotional well-being, although they reported finding no significant differences between insulin- and OHA-treated subgroups of patients with type 2 diabetes mellitus [15].

Regarding the influence of a cohabiting family on stress awareness/coping, our results showed that for males in particular, there was a strong correlation between the presence of a spouse and coping indicators. There was also a strong correlation between the desire for social support in coping with stress and the ADS “Psychological impact of diabetes” subscale. Also, a comparison of males living with or without spouses showed no significant differences on the ADS scale but significant differences in eight of the SCI indicators, with the living with spouse group showing lower emotionality and significantly lower scores than the living without spouses group for social adjustment, acceptance of responsibility, self-control, positive reappraisal, and being methodical and proactive. These results suggest that male patients living with spouses tend to be extremely dependent on their spouses. SCI indicator scores for females, on the other hand, showed no significant differences between those living with/without spouses, although the ADS “Psychological impact of diabetes” subscale score was significantly lower for females living with their spouses, showing that they suffered less stress. Whereas males showed a strong dependence on their spouses, females showed only low dependence on their spouses, although they felt greater stress when living with non-spouse family members than with their spouses. The HbA1c level of Japanese patients with type 2 diabetes mellitus has been shown to be significantly lower for patients under the age of 60 receiving support from families than for those not receiving such support, with males who receive nutritional support rather than advice and encouragement showing particularly better glycemic control [16]. These results suggest that, in addition to gender differences, the division of roles in traditional Japanese culture, with females for the most part preparing meals and otherwise caring for family members, also influences the relationship between spouses and the stress awareness/coping of males. In our recent study, we found that patient empowerment was influenced by the number of disease-related symptoms, age, and gender [17]. The results obtained in this study strongly support our previous study. Therefore, gender, age, and family support is important for maintaining the QOL of patients with type 2 diabetes mellitus.

## Conclusions

The results of this study suggest that gender differences and age are closely related to the stress awareness/coping and diet therapy of patients with type 2 diabetes mellitus and that males in particular are strongly dependent on the support of cohabiting spouses. Therefore, patient education regarding stress awareness and coping needs to be tailored to the individual and adequate attention paid to gender differences, age, family environment, and other factors such as stress condition or social status. It is important to take into account gender, age, and family environment to provide patients with an individualized approach to addressing perceived stress and to provide educational programs for coping that can maximize treatment and maintain better, continuous glycemic control.

## Abbreviations

ADS: Appraisal of Diabetes Scale; SCI: Lazarus Type Stress Coping Inventory; HbA1c: Glycated hemoglobin; OHA: Oral hypoglycemic agent.

## Competing interests

The authors declare that they have no competing interests.

## Authors’ contributions

YH lead in developing this manuscript participated in study design, data collection and drafted manuscript. MH and HI participated in data collection and discussion. MN was involved in data editing, statistical analysis, interpretation, manuscript preparation, and provided discussion and advice. KT actively provided discussion and advice. YI supervised YH, was participated in study design, data editing, analysis, manuscript preparation, and also supervised all aspects of this study. All authors read and approved the final manuscript.
